# Utility of the GerdQ questionnaire in detecting gastroesophageal symptoms with RA patients

**DOI:** 10.1186/s41927-024-00442-2

**Published:** 2024-12-23

**Authors:** Yuji Nozaki, Kazuya Kishimoto, Daisuke Tomita, Tetsu Itami, Chisato Ashida, Koji Kinoshita, Itaru Matsumura

**Affiliations:** Department of Hematology and Rheumatology, Kindai University Faculty of Medicine, Osaka-Sayama, Osaka, 589-8511 Japan

**Keywords:** Rheumatoid arthritis, MTX, Adherence, Patient-reported outcomes, Disease activity

## Abstract

**Objective:**

Rheumatoid arthritis (RA) affects multiple organ systems, including the esophagus. Moreover, one of the major side effects of methotrexate (MTX) is gastrointestinal disorders, which are said to affect medication adherence. We investigated the rate of MTX use, dosage, and the use of glucocorticoids (GCs) and oral non-steroidal anti-inflammatory drugs (NSAIDs) in relation to gastroesophageal (GE) symptoms, and whether they influence RA disease activity.

**Methods:**

This study utilized the GerdQ questionnaire to analyze the influence of GE symptoms on RA disease activity and medication adherence. A total of 558 RA patients participated. On the day of the GerdQ questionnaire, data on age, gender, disease duration, RA disease activity, lab results, and lifestyle factors such as smoking history and alcohol consumption were recorded. Detailed drug information on conventional synthetic DMARDs (csDMARDs), biologic/targeted synthetic DMARDs (b/tsDMARDs), glucocorticoids, and NSAIDs were extracted from medical records. Propensity score matching adjusted patient background characteristics.

**Results:**

Before matching, patients with moderate to high disease activity had higher GE symptoms (12.7% vs. 25.6%). After matching, higher GerdQ scores were correlated with increased tender joint counts 28 (TJC28) and worse visual analog scale (VAS) scores. Oral MTX usage was similar, but the dosage was significantly lower in the group with higher GerdQ scores (51.4% vs. 50.8% and 7.7 ± 2.4 mg/wks vs. 6.5 ± 2.6 mg/wks, *p* < 0.05*).

**Conclusions:**

GE symptoms significantly impact MTX treatment and patient-reported outcomes such as TJC28 and VAS in RA disease activity, highlighting their importance in RA treatment strategies. For clinicians, the study’s results will emphasize the importance of monitoring and managing GE symptoms in RA patients, particularly those on MTX therapy. Furthermore, the data could provide a basis for future studies that explore targeted interventions to mitigate GE symptoms and enhance medication adherence, potentially improving RA outcomes.

## Introduction

Rheumatoid Arthritis (RA) is one of the most common autoimmune diseases, affecting approximately from 0.25 to 1% of the population and causing irreversible cartilage and joint damage leading to disability [1]. RA is a systemic autoimmune disease that affects not only joints but also multiple organ systems. While there have been reports on the relationship between RA disease activity and cardiovascular, lung, and kidney injury [2–4], there is limited information on its impact on gastroesophageal (GE) symptoms [5]. GE symptoms in RA are underexplored, though their potential impact on medication adherence, especially with commonly prescribed drugs like methotrexate (MTX), is increasingly recognized.

MTX is a key drug for RA management and is used as a first-line therapy, playing a crucial role in rheumatological treatment guidelines and recommendations [6]. Oral MTX is the most prescribed treatment for RA and has long been used in clinical practice, with evidence that higher doses improve clinical outcomes [7]. However, with the dose increasing, oral MTX have shown bioavailability variations and frequent GE adverse events (AEs), which limit optimal use [8]. GE symptoms related to MTX, such as nausea, vomiting, and abdominal pain are the most common AEs, along with hair loss, stomatitis, diarrhea, and hepatotoxicity [9]. Folic acid supplementation can alleviate these AEs, but many patients discontinue treatment, adversely affecting disease management and quality of life [10]. This study offers a novel contribution by focusing on the comprehensive assessment of GE symptoms and examining how they influence not only medication adherence but also disease activity. Additionally, the potential for GE symptoms to serve as a predictive factor for treatment noncompliance is a significant and underreported issue in RA care. By providing new data on these symptoms’ prevalence and consequences, this study could guide both clinicians and researchers in tailoring RA treatment strategies more effectively.

## Methods

### Patients

RA patients who were treated with RA at the Department of Hematology and Rheumatology of Kindai University School of Medicine (Osaka, Japan) were enrolled. Age and duration of disease on the day the GerdQ questionnaire was obtained [11], and all details regarding conventional synthetic disease-modifying anti-rheumatic drugs (csDMARDs) and biologics/targeted synthetic disease-modifying anti-rheumatic drugs (b/tsDMARDs) used to treat RA were identified and analyzed from medical records. There was no history of the patients with cancer and ulcer in gastric and duodenum within 3 months. Information was also collected on the presence of comorbidities such as hypertension, dyslipidemia, diabetes, cerebrocardiovascular diseases, and any lung disease as well as on smoking status (–; none, +; 20 cigarettes per day) and alcohol consumption (–; none, +; 4 drinks per day). Patients also took the necessitated oral non-steroidal anti-inflammatory drugs (NSAIDs) (–; none, +; 3 times per week), MTX (–; none, +; 1 mg per week), glucocorticoids (GCs) (–; none, +; 1 mg per day), and proton pump inhibitors (PPIs), potassium competitive acid blockers (P-CAB), histamine type-2 receptor antagonist (H2RA), mucosal protectants (–; none, +; everyday) at a stable dose within 3 months. The presence of active ulcer was not permitted, nor was continuous use of pre-specified concomitant medications including PPIs, P-CAB, H2RA, mucosal protectants. Any patients with cognitive impairment or other conditions that prevent reliable self-reporting are excluded. To be eligible for the present study, the patient’s diagnosis of RA had to be based on the American College of Rheumatology (ACR) criteria [12] before 2009 and the 2010 ACR and the European League Against Rheumatism classification criteria [13] after 2010.

### GerdQ

This was a cross-sectional study conducted and involved the evaluation of RA patients with and without GE symptoms using GerdQ to assess the impact of these symptoms on RA disease activity and treatment adherence in 2023. Based on the results of the Diamond study [11], GerdQ is a questionnaire comprising six items. It comprises four positive predictors of GERD: heartburn and regurgitation (the two characteristic symptoms of GERD, according to the Montreal definition), sleep disturbance because of these two reflux symptoms and use of over-the-counter medication in addition to that prescribed (found to be positive predictors in the Diamond study) and two negative predictors of GERD, abdominal pain and nausea [11]. The GerdQ questions are derived from the Reflex Disease Questionnaire, the GE Symptom Rating Scale and the gastroesophageal reflex disease Impact Scale. In the current study, patients were asked to recall symptoms present during the last 7 day and complete the questionnaire. The sum of the scores of the six questions was defined as the GerdQ score, ranging from 0 to 18. Of those cut-offs, 8 has the highest specificity (71.4%) and sensitivity (64.6%) and, consequently, it is proposed as the cut-off when testing for GERD [11]. A GerdQ score of 8 or above was defined as the high GerdQ score group, while a score below 8 was defined as the low GerdQ score group.

### Clinical assessment and laboratory data

The assessment for the RA disease activity was the Clinical Disease Activity Index (CDAI) [14] in the 558 RA patients. The parameters included the proportion of patients with a complete set of data of the CDAI, and tender joint count 28 (TJC28) and swollen joint count 28 (SJC28) (both of which were determined by the patient’s treating physician), the patient and doctor-visual analogue scale (Pt and Dr-VAS). The patients’ physical function at baseline was evaluated based on their health assessment questionnaire disability index (HAQDI) score [15]. On the day of GerdQ questionnaire collection, laboratory parameters including C-reactive protein (CRP) (mg/dL), erythrocyte sedimentation rate (ESR) (mm/h), rheumatoid factor (RF) (U/mL), aspartate aminotransferase (AST), alanine aminotransferase (ALT), platelet count, Fibrosis-4 (FIB-4) index [16], estimated glomerular filtration rate (eGFR), and the prevalence of chronic kidney disease (CKD) [17], were assessed. The FIB-4 index is a clinical tool for assessing the degree of liver fibrosis; the FIB-4 uses common laboratory results to calculate values that help physicians determine a patient’s risk of liver fibrosis [18]. As a risk category, FIB-4 < 1.45 is defined as low risk of liver fibrosis, 1.45 ≤ FIB-4 ≤ 3.25 as intermediate risk of liver fibrosis, and FIB-4 > 3.25 as requiring further evaluation is defined as high risk of liver fibrosis and requires further confirmation and treatment. CKD was defined as eGFR < 60 mL/min/1.73 m² for at least 3 months, and patients with eGFR < 60 on the day the GerdQ questionnaire was obtained and at least 3 months prior were defined as CKD patients [19]. Each patient’s anti-citrullinated protein antibody (ACPA) (U/mL) was measured on the diagnosis date or the nearest examination dates. The Steinbrocker staging on X-ray evaluation and class classification for RA based were recorded by the patient’s treating physician.

### Statistical analysis

The results of the analyses presented in this study in patients with RA can be summarized as follows: Differences between distributions of continuous data were compared using Mann-Whitney U test for comparisons Chi-square test was used to assess differences in categorical data. P value less than 0.05 were considered significant.

To identify potential factors associated with moderate to high disease activity, univariable logistic regression analysis was performed using baseline variables, including age, sex, the positivity off RF, MTX and GCs use, the proportion of previously failed > 1 bDMARDs, high GerdQ score group (defined as GerdQ ≥ 8) and eGFR < 60 mL/min/1.73 m², FIB-4 index, and comorbidities such as any lung and cerebrocardiovascular diseases. Odds ratios (ORs) and 95% confidence intervals (CIs) were calculated for each variable. Variables with a *p* < 0.1 in univariable analysis were included in the multivariable analysis. To identify independent predictors of moderate to high disease activity, multivariable logistic regression analysis was conducted. Variables included in the final model were selected based on clinical relevance and results from univariable analysis. Adjusted ORs and 95% CIs were calculated, with statistical significance set at *p* < 0.05. We also adjusted by using the propensity scores from a logistic regression model with the following variables: age, gender, body mass index (BMI), duration of RA, history of smoking and drinking alcohol, current medication of PPI or P-CAB, the positivity and titers of RF and ACPA, and then, we matched these scores one by one using nearest-neighbor methods without replacement, and no caliper width. Through the matching procedure for propensity scores, the two groups showed similar distributions of propensity scores, indicating that the differences in covariates between the two groups were minimized. For continuous variables, summary statistics of mean ± standard deviation (SD) or median and interquartile range (IQR) are presented as appropriate. Categorical variables are presented as percentages; P levels of less than 0.05 were considered significant. Statistical analysis was performed using statistical analysis software (GraphPad Prism, GraphPad Software, San Diego, CA) and JMP statistical software (SAS Institute, Cary, NC).

## Results

### Patient characteristics

The demographic characteristics of the study participants are shown in Table 1. A total of 558 patients with RA were analyzed. We divided the patients into two groups based on their CDAI: the LDA + Remission group (low disease activity to remission) and MDA + HDA (moderate to high disease activity) groups. The CDAI scores were median 1.6 [0.2–4.5] for the LDA + Remission group and median 13.1 [11.7–19.9] for the MDA + HDA group. Age distribution showed no significant differences between the two groups. However, the proportion of females was significantly higher in the MDA + HDA group compared to the LDA + Remission group (86.1% vs. 72.3%, *p* < 0.05*). Additionally, the duration of the disease was longer in the MDA + HDA group (median 96.0 months [67.0–204.0]) compared to the LDA + Remission group (median 79.5 months [38.0–158.5]). The BMI was also lower in the MDA + HDA group (mean 20.6 ± 3.7) than in the LDA + Remission group (mean 22.6 ± 3.7).

**Table 1:** The 558 rheumatoid arthritis patients’ baseline clinical and laboratory data and treatment information

	All patients *n* = 558	CDAI LDA + Remission *n* = 452	CDAI MDA + HDA *n* = 106
Age, yes	65.7 ± 13.5	65.5 ± 13.5	64.7 ± 12.9
Female (%)	73.6	72.3	86.1*
Disease duration, months [IQR]	82.5 [40.0–163.0]	79.5 [38.0–158.5]	96.0 [67.0–204.0]*
Body Mass Index	22.4 ± 3.7	22.6 ± 3.7	20.6 ± 3.7**
Rheumatoid factor (%)/titers [IQR]	55.6, 20.0 [5.3–68.8]	56.8, 20.0 [6.0–67.0]	59.5, 29.5 [5.0-182.0]*
Anticitrullinated peptide antibody (%)/titers [IQR]	57.7, 16.9 [0.0-125.4]	57.8, 16.1 [0.0-121.6]	65.1, 39.2 [0.0-196.5]
C-reactive protein, mg/dL [IQR]	0.1 [0.0–0.3]	0.1 [0.0–0.3]	0.3 [0.0–1.4]*
Erythrocyte sedimentation rate, mm/hr [IQR]	14.0 [8.0–27.0]	13.0 [8.0–26.0]	24.0 [10.0–39.0]*
Tender joints, range 0–28 [IQR]	0.0 [0.0–0.0]	0.0 [0.0–0.0]	3.0 [2.0–6.0]**
Swollen joints, range 0–28 [IQR]	0.0 [0.0–1.0]	0.0 [0.0–1.0]	4.0 [2.0–7.0]**
Patient visual analogue scale, 0–100 mm [IQR]	13.0 [2.0–36.0]	11.0 [1.0–30.0]	62.0 [50.0–77.0]**
Doctor visual analogue scale, 0–100 mm [IQR]	5.0 [1.0–16.3]	4.0 [1.0–13.0]	38.0 [22.0–52.0]**
Clinical Disease Activity Index [IQR]	2.1 [0.3–5.3]	1.6 [0.2–4.5]	13.1 [11.7–19.9]**
High/ Moderate/ Low/ Remission	0.5/7.4/33.5/58.6	0.0/0.0/36.4/63.6	7.0/93.0/0.0/0.0
Medication			
PPI or P-CAB (%)	43.7	42.8	48.8
Histamine H2 receptor antagonist (%)	5.0	4.9	7.0
Gastric mucosa protectant (%)	16.3	15.4	27.9*
Non-steroidal anti-inflammatory drugs (%)	20.6	19.2	34.9*
Cyclooxygenase-II inhibitor (%)	7.4	6.7	16.3*
Others (%)	13.2	12.5	18.6*
csDMARDs			
MTX (%), mg/week	52.4, 4.2 ± 4.0	54.0, 3.9 ± 4.0	51.2, 4.8 ± 4.4
Folic acid (%), mg/week	52.1, 4.6 ± 0.3	53.8, 4.6 ± 0.3	51.0, 4.6 ± 0.3
Salazosulfapyridine (%)	31.0	28.5	46.5*
Iguratimod (%)	38.2	38.5	34.9
Bucillamine (%)	2.9	2.8	2.3
Tacrolimus (%)	5.8	6.3	0.0*
bDMARDs	36.0	35.8	39.5
bDMARDs naive (%)	28.1	28.1	23.3
Previously failed > 1 bDMARDs (%)	16.2	15.6	30.3*
TNFi/IL-6Ri/ CTLA4-Ig (%)	12.3/21.7/12.3	12.3/21.3/12.0	11.6/22.7/14.3
tsDMARDs (%)	9.4	8.7	16.3
Glucocorticoid (%), mg/day [IQR]	22.0, 0.0 [0.0–0.0]	21.0, 0.0 [0.0–0.0]	32.6, 0.0 [0.0-2.5]
Comorbidities (%)			
Hypertension (%)	29.7	29.1	28.6
Dyslipidemia (%)	25.5	25.7	19.1
Diabetes (%)	12.3	12.4	9.5
Cerebrocardiovascular diseases (%)	9.5	4.3	11.9*
Any Lung diseases (%)	1.5	1.3	1.4
Current/Past Smoking (%)	10.6/32.1	10.3/32.7	11.6/25.6
Current/Past Alchol (%)	20.1/58.5	20.3/59.4	18.5/42.8
Steinbrocker Stage	1.8 ± 1.1	1.7 ± 0.1	2.3 ± 0.2*
Steinbrocker Class	1.4 ± 0.7	1.4 ± 0.7	1.8 ± 0.2
HAQDI, range 0–3 [IQR]	0.1 [0.0–0.6]	0.1 [0.0–0.5]	1.0 [0.4–1.5]

Values are median [25th–75th centiles] or mean (SD), unless otherwise indicated. PPI: proton pump inhibitor, P-CAB: potassium-competitive acid blocke, csDMARDs: conventional synthetic disease-modifying antirheumatic drugs, MTX: methotrexate, bDMARDs: biological disease-modifying antirheumatic drugs, TNFi: tumor necrosis factor inhibitor, IL-6Ri: interleukin-6 receptor inhibitor, CTLA-Ig: cytotoxic T-lymphocyte-associated protein 4-immunoglobulin, tsDMARDs: targeted synthetic disease-modifying antirheumatic drugs, HAQDI: health assessment questionnaire disability index, IQR: interquartile range, *p < 0.05, **p < 0.01

The percentage of patients using NSAIDs was higher in the MDA + HDA group (34.9%) compared to the LDA + Remission group (19.2%). While the use of csDMARDs, including MTX, folic acid, and the patients with naïve bDMARD, showed no significant differences between the two groups, there was a trend toward greater use of tsDMARDs and GCs in the MDA + HDA group. Notably, the proportion of patients who had previously failed treatment with one or more bDMARDs was significantly higher in the MDA + HDA group compared to the LDA + Remission group (15.6% vs. 30.3%, *p < 0.05).

**Fig. 1:**
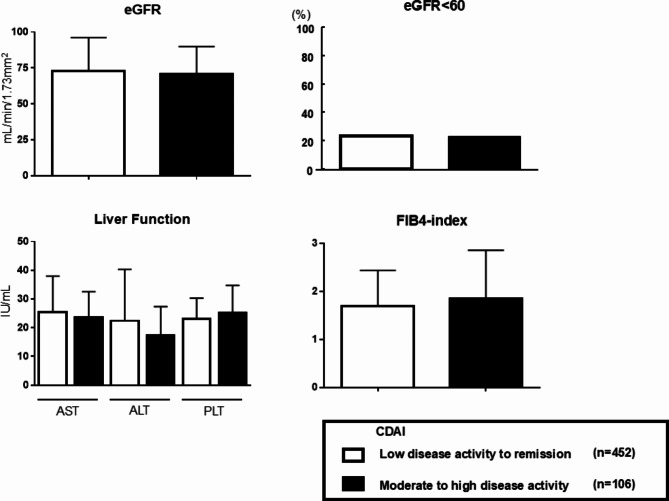
Renal and hepatic symptoms in RA disease activity in 518 patients before propensity score matching Evaluation of eGFR (estimated glomerular filtration rate) and the rate of chronic kidney disease in renal function, and the liver function of AST, ALT, PLT, and the FIB4 index according to CDAI. Abbreviation: RA; rheumatoid arthritis, CDAI: Clinical Disease Activity Index, eGFR: estimated glomerular filtration rate, CKD: chronic kidney disease, AST: aspartate aminotransferase, ALT: alanine aminotransferase, PLT: platelets, FIB4-index; fibrosis index based on four factors

In terms of comorbidities, no significant difference in the prevalence of hypertension, dyslipidemia, diabetes, and any lung diseases including interstitial pneumonia and chronic obstructive pulmonary disease were observed between the two groups. However, the prevalence of cerebrocardiovascular disease was significantly higher in the MDA + HDA group compared to the LDA + Remission group (11.9% vs. 4.3%, *p < 0.05). Regarding lifestyle habits, there were no differences between the groups in terms of smoking or alcohol consumption. However, the Steinbrocker stage, indicating disease severity, was significantly higher in the MDA + HDA group compared to the LDA + Remission group (mean 2.3 ± 0.2 vs. 1.7 ± 0.1, p*<0.05). In Fig. 1, renal function assessed by eGFR and the proportion of CKD patients (eGFR < 60 mL/min/1.73 m²), as well as liver function evaluated using the FIB4 index calculated from AST, ALT, and PLT counts, were analyzed across both groups. There were no differences between the groups in terms of eGFR values and the proportion of CKD patients. Similarly, the FIB-4 index showed no significant difference between the groups.

### RA disease activity and GE symptoms

**Fig. 2:**
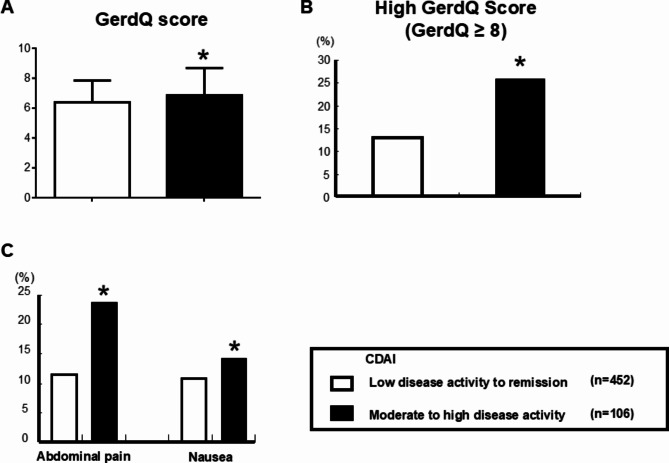
Gastroesophageal symptoms on RA disease activity using the GerdQ questionnaire in 518 patients before propensity score matching (**A**) GerdQ scores for the RA low disease activity to remission group (*n* = 412) and the moderate to high disease activity group (*n* = 106). (**B**) Percentage of the RA low disease activity to remission group and the moderate to high disease activity group with high GerdQ scores. (**C**) Gastric symptoms on the GerdQ questionnaire in the low disease activity to remission group and the moderate to high disease activity group. Comparisons between the two groups’ mean levels and categorical data were analyzed using the Mann-Whitney and Chi-squared test (*p < 0.05) Abbreviation: RA; rheumatoid arthritis, CDAI; Clinical Disease Activity Index

Next, we analyzed GE symptoms using the GerdQ questionnaire in relation to RA disease activity (Fig. 2). Figure 2A showed the results of the analysis of GerdQ scores according to CDAI as RA disease activity. The scores were significantly higher in the MDA + HDA group compared to the LDA + Remission group (mean 6.4 ± 1.4 vs. 6.9 ± 1.8, p*<0.05), and the proportion of high GerdQ score group (defined as GerdQ ≥ 8) was also increased significantly (12.7% vs. 25.6%, p*<0.05) (Fig. 2B). Regarding the questionnaire　on abdominal pain and nausea in the GerdQ, the proportion of patients experiencing these issues was significantly higher in the MDA + HDA group compared to the LDA + Remission group (11.3% vs. 23.8%, 10.9% vs. 14.3%, p*<0.05) (Fig. 2C).

### Factors associated with moderate to high disease activity

Table 2 summarized the results of univariate and multivariate logistic regression analyses conducted to identify factors associated with the MDA + HDA. In the univariate analysis, several factors showed significant associations with the MDA + HDA. Female sex (OR: 2.4, 95% CI: 1.0-5.7, *p < 0.05), previously failed > 1 bDMARDs (OR: 2.5, 95% CI: 1.3–5.1, *p < 0.05), and high GerdQ score group (defined as GerdQ ≥ 8) (OR: 2.4, 95% CI: 1.1–4.9, *p < 0.05) were significantly associated with increased odds of the MDA + HDA. GCs use also demonstrated a trend towards significance (OR: 1.8, 95% CI: 0.9–3.6, *p* = 0.09). After adjusting for relevant variables in the multivariate analysis, GCs use (adjusted OR: 2.2, 95% CI: 1.1–4.6, *p < 0.05), having one or more previously ineffective bDMARDs (adjusted OR: 2.3, 95% CI: 1.1–4.9, *p < 0.05), and high GerdQ score group (adjusted OR: 2.2, 95% CI: 1.1–4.8, *p < 0.05) remained independent predictors of MDA + HDA groups. Other factors, including age, sex, the positivity of RF, MTX use, eGFR < 60 mL/min/1.73 m², FIB-4 index, lung disease, and cerebrovascular disease, did not show significant associations. These findings suggested the importance of specific risk factors in predicting the MDA + HDA.

**Table 2:** Univariate and multivariate analysis of factors associated with moderate to high disease activity

	Univariate		Multivariate	
	OR (95% CI)	*p*-value	OR (95% CI)	*p*-value
Age, yes	1.0 (1.0-1.1)	0.68		
Female (%)	2.4 (1.0-5.7)	0.04	-	-
Rheumatoid factor (%)	0.9 (0.6–1.4)	0.72		
MTX (%)	1.1 (0.8–1.5)	0.72		
Glucocorticoid (%)	1.8 (0.9–3.6)	0.09	2.2 (1.1–4.6)	0.03*
Previously failed > 1 bDMARDs (%)	2.5 (1.3–5.1)	0.01	2.3 (1.1–4.9)	0.02*
High GerdQ score group (defined as GerdQ ≥ 8) (%)	2.4 (1.1–4.9)	0.03	2.2 (1.1–4.8)	0.03*
eGFR < 60 mL/min/1.73 m² (%)	1.0 (0.8–1.2)	0.92		
FIB-4 index	0.8 (0.6–1.2)	0.31		
Any Lung diseases (%)	1.1 (0.4–2.9)	0.85		
Cerebrocardiovascular diseases (%)	0.9 (0.8-1.0)	0.06		

P-values were determined by univariate or multivariate logistic regression analysis. OR: odds ratio, Fibrosis-4 index: (FIB-4) index, eGFR: estimated glomerular filtration rate, bDMARDs: biologics disease-modifying antirheumatic drugs, MTX: methotrexate, *p < 0.05

### Propensity matching of backgrounds in RA patients

We retrospectively analyzed the backgrounds of RA patients before and after propensity score matching, dividing them into high and low GerdQ score groups, with the results shown in Table 3. Before propensity score matching, when comparing the low and high GerdQ score groups, it was observed that the latter group tended to have older patients with longer disease durations. Similarly, the high GerdQ score group had significantly higher HAQ scores compared to the low GerdQ score group. Given the imbalance between these two groups, interpreting the results in terms of disease activity assessment parameters such as CDAI could be challenging. Therefore, before conducting statistical analysis, propensity score matching was performed to adjust for these differences. As a result of the adjustment through propensity score matching, no differences were observed between the two groups in terms of patient age, disease duration, comorbid cerebrocardiovascular diseases, and HAQDI. In both groups, the proportion of patients with a history of failure to respond to more than one bDMARD also showed no significant differences between the low and high GerdQ score groups, before and after propensity score matching.

**Table 3:** The 558 rheumatoid arthritis patients’ baseline clinical and laboratory data and treatment information

	Before propensity matching			After propensity matching	
All patients *n* = 558	Low GerdQ score *n* = 482	High GerdQ score *n* = 76		Low GerdQ score *n* = 67	High GerdQ score *n* = 68
Age	65.4 ± 0.6	68.0 ± 1.6		65.5 ± 1.7	68.0 ± 1.7
Female (%)	72.6	80.0		82.4	82.4
Disease duration, months	63.5 [38.3–152.0]	84.0 [37.0–162.5]		81.0 [40.0–164.0]	84.0 [37.0–165.8]
RF (%), titers	55.1, 20.0 [5.0-66.3]	59.5, 24.0 [6.0-77.3]		57.4, 20.0 [6.0-67.8]	61.8, 24.0 [6.0-77.5]
ACPA (%), titers	58.6, 17.3 [0.0-124.8]	54.7, 16.2 [0.0-131.1]		58.8, 18.9 [0.0-183.3]	55.9, 16.1 [0.0-126.8]
Medication					
PPI or P-CAB (%)	43.7	48.0		46.0	49.2
Histamine H2 receptor antagonist (%)	4.8	6.7		3.2	6.4
Gastric mucosa protectant (%)	16.8	13.3		19.1	12.7
NSAIDs (%)	20.5	21.3		23.8	17.5
COX-II inhibitor/Others (%)	7.5/13.0	6.7/13.6		12.7/11.1	4.8/12.7
csDMARDs (%)					
SASP/IGU/BUC/TAC (%)	30.6/38.0/2.9/6.2	33.3/40.0/2.7/2.8		34.9/33.3/4.8/9.8	34.9/40.0/3.2/3.3
bDMARDs (%)	57.0	60.7		58.6	60.6
Previously failed > 1 bDMARDs (%)	14.7	20.0		12.9	23.1*
TNFi/IL-6Ri/CTLA4-Ig (%)	12.5/32.5/12.0	14.7/32.0/14.0		8.8/35.2/14.6	14.7/30.3/15.6
tsDMARDs (%)	12.1	10.9		7.0	15.1
Body Mass Index	22.3 ± 3.8	23.0 ± 3.5		22.8 ± 3.9	22.9 ± 3.6
Comorbidities (%)					
Hypertension, (%)	26.6	21.3		28.6	24.6
Dyslipidemia (%)	26.2	21.3		31.4	21.5
Diabetes (%)	12.6	10.7		15.7	9.2
Cerebrocardiovascular diseases (%)	5.2	5.3		7.1	6.2
Any Lung diseases (%)	1.4	1.2		1.1	1.3
Smoking: Current/Past (%)	10.8/31.9	9.3/33.3		9.7/24.3	10.8/33.9
Alchol: Current/Past (%)	21.1/23.2	14.7/27.0		13.5/25.0	11.7/28.4
Steinbrocker Stage/Class	1.8 ± 0.1/1.4 ± 0.7	1.7 ± 0.2/1.4 ± 0.2		1.8 ± 1.1/1.4 ± 0.9	1.6 ± 1.2/1.3 ± 1.1
HAQDI, range 0–3	0.1 [0.0–0.6]	0.4 [0.0–1.0] **		0.2 [0.0–0.3]	0.3 [0.2–0.5]

PPI: proton pump inhibitor, P-CAB: potassium-competitive acid blocke, NSAIDs: non-steroidal anti-inflammatory drugs, COX: cyclooxygenase, csDMARDs: conventional synthetic disease-modifying antirheumatic drugs, SASP: Salazosulfapyridine, IGU: Iguratimod, BUC: Bucillamine, TAC: Tacrolimus, bDMARDs: biological disease-modifying antirheumatic drugs, TNFi: tumor necrosis factor inhibitor, IL-6Ri: interleukin-6 receptor inhibitor, CTLA-Ig: cytotoxic T-lymphocyte-associated protein 4-immunoglobulin, tsDMARDs: targeted synthetic disease-modifying antirheumatic drugs, GCs: glucocorticoids, HAQDI: health assessment questionnaire disability index. We calculated the propensity scores from a logistic regression model with the following variables: age, gender, body mass index (BMI), duration of RA, history of smoking and drinking alcohol, current medication of NSAIDs, GCs, PPI or P-CAB, the positivity of RF and ACPA, and then, we matched these scores one by one using nearest-neighbor methods without replacement, and no caliper width. Through the matching procedure for propensity scores, the two groups showed similar distributions of propensity scores, indicating that the differences in covariates between the two groups were minimized. Summary statistics of the mean ± standard deviation (SD) or the median and interquartile range (IQR) are presented for continuous variables as appropriate. Categorical variables are presented as percentages. Comparisons between independent means were analyzed using the Mann-Whitney test and paired *t*-test (*p < 0.05, **p < 0.01). A GerdQ score of 8 or above was defined as the high GerdQ score group, while a score below 8 was defined as the low GerdQ score group

The baseline usage rate of tsDMARDs tended to be higher in the high GerdQ score group compared to the low GerdQ score group, but this difference was not significant. Regarding comorbidities, no significant differences were observed between the two groups in the prevalence of hypertension, dyslipidemia, diabetes, cerebrocardiovascular diseases, and any lung diseases. Statistical analysis proceeded on these two groups after adjustment for distribution via propensity score matching.

### Clinical disease activity after propensity matching

**Fig. 3:**
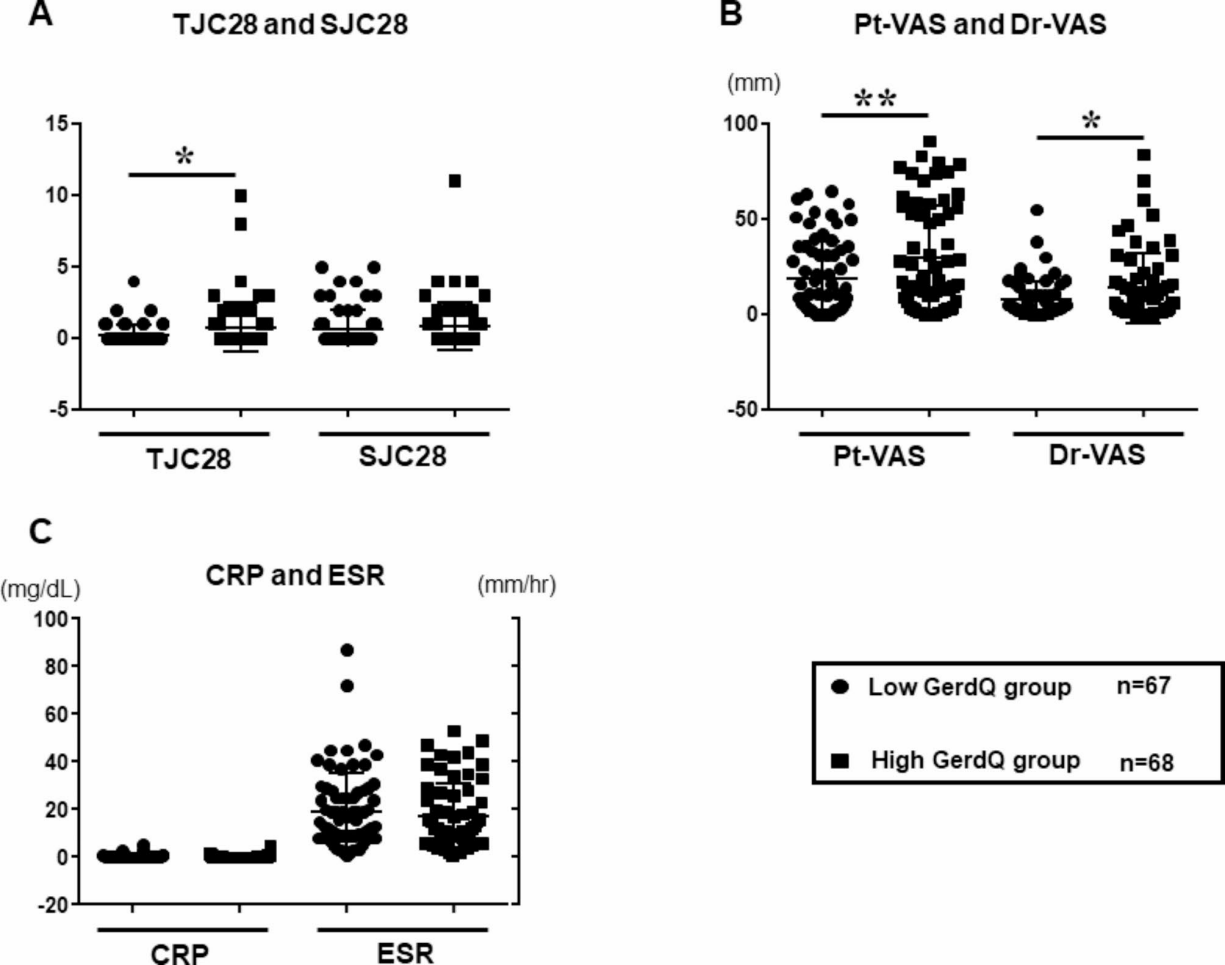
Patient-reported outcomes and inflammatory markers in RA patients after propensity matching (**A**) TJC and SJC 28 in patients with the low and high GerdQ score. (**B**) Pt and Dr-VAS with the low and high GerdQ score. (**C**) CRP levels and ESR in patients with the low and high GerdQ score. Comparisons between the two groups’ mean levels were analyzed using the Mann-Whitney test (*p < 0.05). Abbreviation: TJC28; tender joint counts 28, SJC28; swollen joint counts 28, Pt and Dr-VAS; patient and doctor-visual analogue scale, CRP, C-reactive protein, ESR; erythrocyte sedimentation rate

**Fig. 4:**
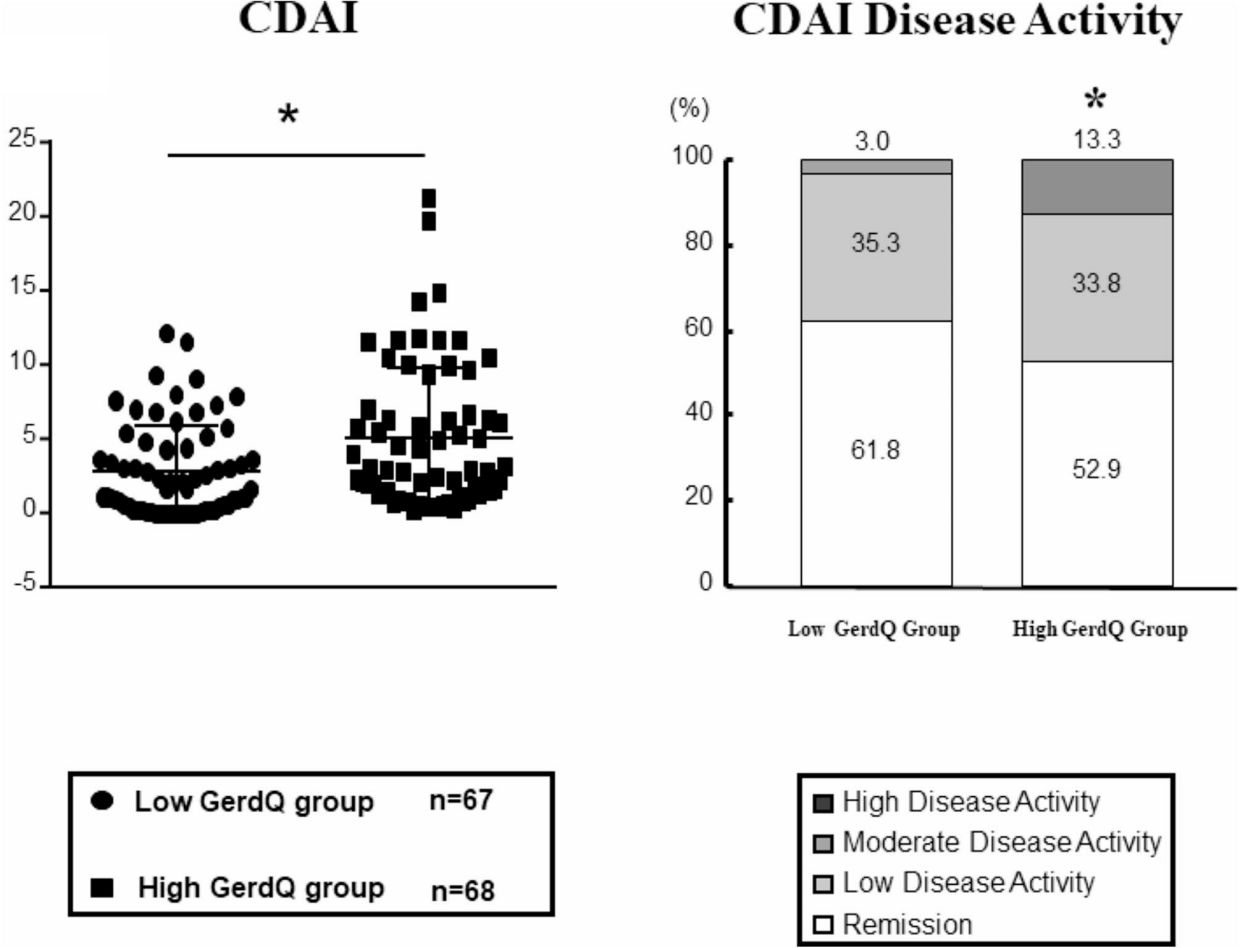
GerdQ Scores on RA Disease Activity Investigation of CDAI scores and CDAI Disease Activity by the low and high GerdQ score group. Comparisons between the two groups’ mean levels and categorical data were analyzed using the Mann-Whitney and Chi-squared test (*p < 0.05). Abbreviation: RA; rheumatoid arthritis, CDAI; Clinical Disease Activity Index

Figure 3 illustrates the clinical disease activity assessment items in the two groups divided by low and high GerdQ scores. Significant differences in TJC28 were observed between the low and high GerdQ score groups (mean 0.2 ± 0.1 vs. 0.8 ± 1.7, *p < 0.05). It was also observed that components of patient-reported outcomes (PROs), such as the Pt-VAS (median 9.5 [2.0–33.3] vs. 17.0 [5.5–54.5]), and Dr-VAS (median 4.0 [1.0–10.0] vs. 6.5 [1.0–17.8], p*<0.05 and p**<0.01), increased significantly in the high GerdQ score group compared to the low GerdQ score group (Fig. 3A and B). Conversely, there were no significant differences between the two groups in terms of the number of SJC28 and inflammatory biomarkers such as CRP and ESR (Fig. 3C). Figure 4 presents the CDAI scores for the two groups of patients with high and low GerdQ scores. The CDAI score was significantly higher in the high GerdQ score group compared with the low GerdQ score group (mean 2.7 ± 0.4 vs. 4.6 ± 0.6, p*<0.05). Remission and low disease activity rates (61.8% vs. 52.9% and 35.3% vs. 33.8%, respectively) did not differ between the two groups. However, the proportion of patients with moderate CDAI disease activity was significantly higher in the high GerdQ score group compared to the low GerdQ score group (3.0% vs. 13.3%, p*<0.05).

### The influences of gastroesophageal reflux disease symptoms on RA treatment

**Fig. 5:**
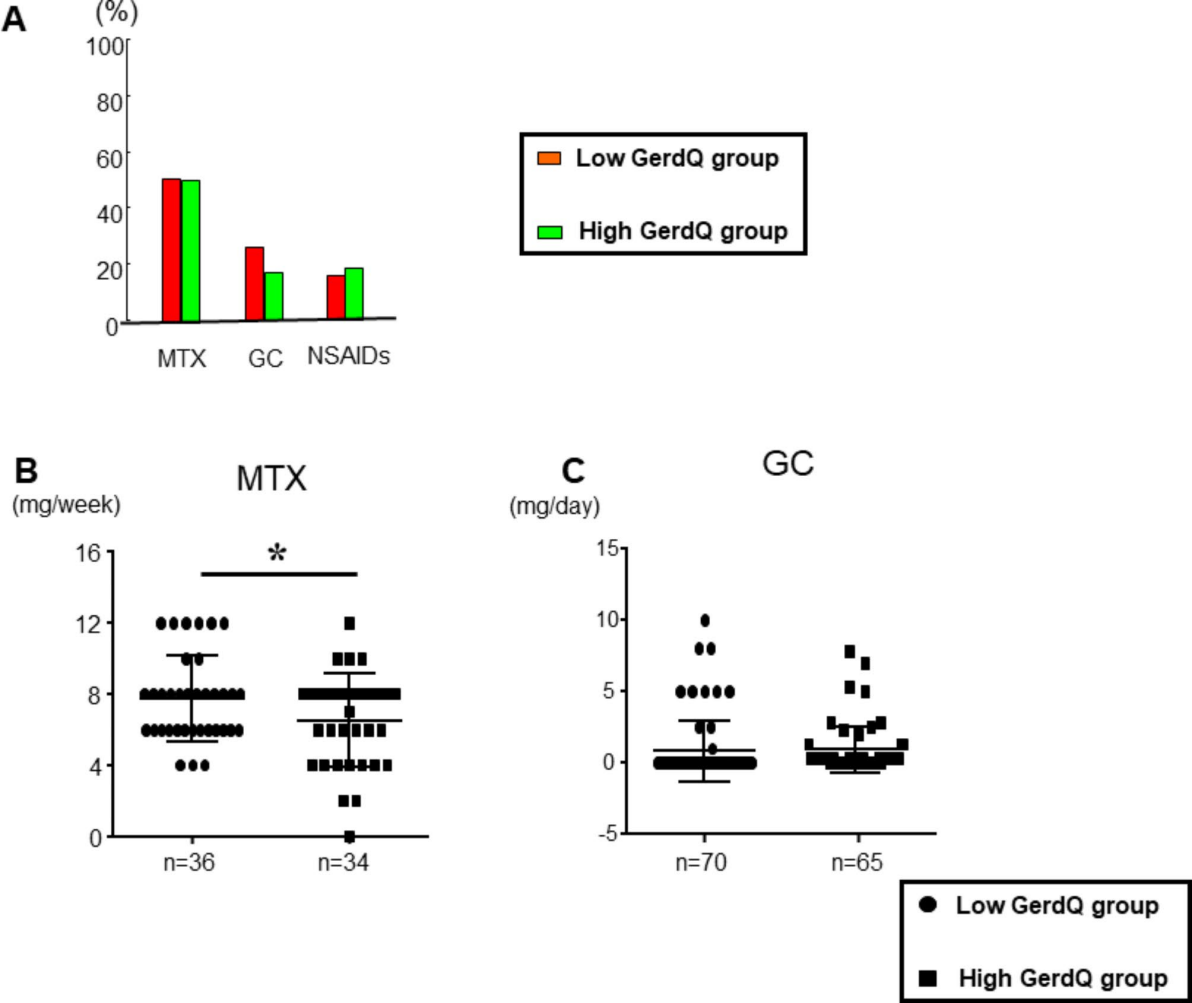
GerdQ scores on RA medication adherence Adherence with RA medications, categorized by the low and high GerdQ score group. (**A**) MTX, GCs, and NSAIDs adherence rate in patients with the low and high GerdQ score. (**B** and **C**) MTX and GCs oral dose in patients with the low and high GerdQ score. Comparisons between the two groups’ mean levels and categorical data were analyzed using the Mann-Whitney and Chi-squared test (*p < 0.05) Abbreviation: RA; rheumatoid arthritis, MTX; methotrexate, GCs; glucocorticoids, NSAIDs; non-steroidal anti-inflammatory drugs

Figure 5 demonstrates adherence to RA medications, categorized by high and low GerdQ score groups. Figure 5A shows the medication usage of MTX, GCs, and NSAIDs in the two groups: patients with high GerdQ scores and those with low GerdQ scores (MTX: 51.4% vs. 50.8%; GCs: 25.7% vs. 16.9%; NSAIDs: 15.7% vs. 18.5%). There were no significant differences in the percentage of patients using MTX, GCs, or NSAIDs between the two groups (Fig. 5A). However, the dosage of MTX was significantly lower in patients with high GerdQ scores compared to those with low GerdQ scores (mean 7.7 ± 2.4 mg/wks vs. 6.5 ± 2.6 mg/wks, p*<0.05) (Fig. 5B). Conversely, there was no disparity in GCs dosage between the two groups (mean 0.8 ± 0.3 mg/day vs. 0.6 ± 0.2 mg/day) (Fig. 5C).

## Discussion

This study is the first cross-sectional report from a single-center investigation examining upper gastrointestinal symptoms using the GerdQ questionnaire in 558 patients of RA, assessing their impact on disease activity. In the group with high GerdQ scores, both the TJC28 and VAS were significantly increased compared to those with low GerdQ scores. However, there were no significant differences in inflammatory markers such as CRP and ESR between the two groups. Despite the absence of differences in inflammatory markers, the observed differences in TJC28 and VAS influenced the significant disparity in CDAI scores between the two groups. Examining the impact of GE symptoms on the use of RA medications, there was no significant difference observed between the two groups in the use of GCs or NSAIDs. However, while the rate of MTX usage did not differ between the groups, the oral MTX dosage was significantly lower in the group with high GerdQ score compared to those with low GerdQ score. Furthermore, significant increases in symptoms such as abdominal pain and nausea, not reflected in the GerdQ score, were observed in the patients with high GerdQ score group compared to those with low GerdQ score group. Among RA patients experiencing abdominal pain and nausea, there was a significant decrease in adherence to MTX treatment. Therefore, it is suggested that GE symptoms not only impact PROs and elevate CDAI scores but may also worsen RA disease activity by affecting MTX adherence. It is widely recognized that RA patients frequently experience mood symptoms such as anxiety and depressive symptoms that interfere with their daily lives [20]. According to the current definition of health by the World Health Organization, health is a state of complete physical, mental, and social well-being [21, 22]. Therefore, mood symptoms can have a significant impact on the health status of patients with inflammatory arthritis. Japanese studies have reported a higher prevalence of GE symptoms in RA patients compared to the general population (24.6% vs. 11.5%), and US studies have shown an increased risk of GE symptoms in RA patients younger than 60 years [23, 24]. Anti-rheumatic drugs, particularly NSAIDs, are thought to contribute to the increased burden of GE symptoms in RA patients [25–27].

In our current study, despite normal inflammatory markers, the increase in TJC28 and Pt and Dr-VAS in the group with high GerdQ score compared to those with the low GerdQ score suggests that the impact of GE symptoms on PROs should be sufficiently considered. However, in this study, we did not actually measure mood symptoms in RA patients using tools like the 36-item Short-Form Health Survey　(SF-36) [27], and thus, we could not demonstrate the presence of mood symptoms in RA patients with GE symptoms. In addition to mood symptoms, drug treatment is a factor related to GE symptoms affecting RA disease activity. It is widely known that MTX, a key drug RA treatment, influences the treatment due to GE symptoms such as nausea and abdominal pain. We also demonstrated that patients with moderate to high disease activity group in CDAI had significantly higher rates of abdominal pain and nausea compared to the low activity to remission group. Although the rate of MTX usage was similar between the two groups, the MTX oral dosage was lower in the group with the high GerdQ score compared to those with the low GerdQ score. These results suggest that GE symptoms affected MTX adherence, potentially leading to increased CDAI scores. There were no significant differences between the two groups in NSAIDs and GCs, the risk of conventional GE symptoms. GE symptoms (nausea, vomiting, and abdominal pain) due to MTX administration are the most common, along with others such as hair loss, stomatitis, and hepatotoxicity. Three mechanisms have been hypothesized for MTX-related GE symptoms. As an effect on the gastrointestinal mucosal epithelium, oral and intestinal epithelial cells rapidly renew and are sensitive to MTX regardless of folate deficiency. Over time, the gastrointestinal epithelium becomes sensitized to MTX accumulation, causing nausea, vomiting, and myelosuppression, leading to cytopenia [28, 29]. Regarding the central nervous system, tolerance may arise through binding to adenosine receptors in the central nervous system [30]. The Chemoreceptor Trigger Zone detects emetic substances in the blood, communicating to the vomiting center to induce the vomiting reflex. Hepatotoxicity and central nervous system toxicity are more complex, involving elevated liver enzymes, headaches, and behavioral changes [31]. Some patients develop anticipatory symptoms before MTX intake, presenting symptoms just by thinking about the drug, along with behavioral symptoms like anxiety and irritability. The J-RAPID trial, which combined certolizumab pegol (CZP) with standard MTX treatment for RA patients with an inadequate response to MTX [32], and the C-OPERA trial [33], which rapidly increased MTX dosages in Japanese early RA patients in both the placebo and CZP groups, highlight the importance of understanding MTX’s gastrointestinal AEs. In the J-RAPID trial, RA patients received lower doses of MTX (6–8 mg/week), whereas in the C-OPERA trial, RA patients received higher doses of MTX (12 mg/week) at baseline. Consequently, AEs were approximately 1.3–1.5 times more frequent in the C-OPERA trial. Specifically, the incidence of AEs was higher in the “infections and infestations,” “gastrointestinal symptoms,” and “hepatobiliary and pancreatic symptoms” categories in the C-OPERA trial. These AEs were more common in both the placebo and CZP groups, with no significant difference between the two groups. The increased frequency of AEs in the C-OPERA trial suggests that the higher doses of MTX used in this trial may be associated with the greater incidence of AEs. Particularly, GE symptoms of MTX can necessitate dose reduction or discontinuation. Early detection of GE symptoms through patient interviews can allow for intervention, such as switching to subcutaneous MTX formulations or adding antiemetics, thereby potentially avoiding exacerbation of RA disease activity. We conducted both univariate and multivariate analyses to identify factors influencing RA disease activity. Multivariate analysis revealed that, in addition to GerdQ scores, the use of GCs and two or more bDMARDs were significantly associated with RA disease activity. Propensity score matching analysis showed no association between GCs use and GerdQ scores, however, a higher proportion of patients using two or more bDMARDs was observed in the high GerdQ score group. Furthermore, patients with high GerdQ scores tended to exhibit elevated TJC28 and VAS, suggesting that their current bDMARD therapy may be perceived as ineffective by their physicians. As a result, these patients are more likely to be switched to an alternative bDMARD.

This study has several limitations that should be acknowledged. One significant finding was that the GerdQ score seems to influence the oral MTX dose and reflects RA disease activity. However, the cross-sectional design did not consider the duration of RA treatment, which limits the understanding of the relationship between GE symptoms and MTX dosing. The study included patients at various stages of MTX treatment, those who had just started, those rapidly escalating doses, and those on stable doses for prolonged periods. This lack of temporal analysis limits the ability to capture dynamic changes over time. Future studies should include time-varying analyses or assess the average cumulative MTX dose, especially focusing on patients who did not follow a dose-escalation strategy. This would provide more comprehensive insights into how GerdQ scores affect MTX dosing and treatment outcomes over time. The study also lacked temporal analyses related to the onset of GE symptoms in connection with MTX initiation. Including the total cumulative dose of oral MTX in future analyses would provide a clearer understanding of the dose-response relationship, particularly in terms of side effects. This would help to clarify whether GE symptoms are more prevalent at higher cumulative doses or at certain treatment phases. Second, this study did not assess adherence to all prescribed medications for RA treatment using questionnaires.　However, we analyzed cases in which the prescribed medication amount at each visit aligned with the scheduled visit date for the next appointment, as reported by the physician during patient interviews. Finally, the GerdQ, being a self-reported tool, is subject to recall bias, which may have impacted the accuracy of the data. Patients might have under- or over-reported their symptoms based on memory or subjective interpretation, reducing confidence in the findings. While this limitation does not invalidate the study’s conclusions, future research should complement self-reported data with objective diagnostic tools such as endoscopy or pH monitoring to more accurately assess GE symptoms and their impact on RA disease activity and treatment adherence. In summary, addressing these limitations in future studies will provide a more comprehensive understanding of the relationship between GE symptoms, MTX treatment, and RA disease activity, offering valuable insights for both clinicians and researchers. These limitations are recognized in our study; however, this is the first report to highlight the influence of GE symptoms on RA disease activity and MTX oral dosage, making it an important contribution to future RA treatment strategies.

## Conclusion

For clinicians, the results of this study suggest the need for careful monitoring and management of GERD symptoms in RA patients, particularly those undergoing MTX therapy. Addressing GERD symptoms may help optimize medication adherence and potentially improve disease outcomes, especially in patients experiencing gastrointestinal side effects.

## Data Availability

The datasets used and/or analyzed in the present study are available from the corresponding author on reasonable request.

## Abbreviations

RA: Rheumatoid Arthritis
GE: Gastroesophageal
MTX: Methotrexate
AEs: Adverse events
csDMARDs: Conventional synthetic disease-modifying anti-rheumatic drugs
b/tsDMARDs: Biologics/targeted synthetic disease-modifying anti-rheumatic drugs
NSAIDs: Non-steroidal anti-inflammatory drugs
GCs: Glucocorticoids
PPIs: Proton pump inhibitors
P-CAB: Potassium competitive acid blockers
H2RA: Histamine type-2 receptor antagonist
ACR: American College of Rheumatology
CDAI: Clinical Disease Activity Index
TJC28: Tender joint count 28
SJC28: Swollen joint count 28
Pt and Dr-VAS: The patient and doctor-visual analogue scale
CRP: C-reactive protein
ESR: Erythrocyte sedimentation rate
RF: Rheumatoid factor
AST: Aspartate aminotransferase
ALT: Alanine aminotransferase
FIB-4: Fibrosis-4
eGFR: Estimated glomerular filtration rate
CKD: Chronic kidney disease
ACPA: Anti-citrullinated protein antibody
BMI: Body mass index
SD: Standard deviation
IQR: Interquartile range
PROs: Patient-reported outcomes
CZP: Certolizumab pegol
